# Use of antibiotics for common illnesses among children aged under 5 years in a rural community in Indonesia: a cross-sectional study

**DOI:** 10.1186/s41182-019-0173-6

**Published:** 2019-07-18

**Authors:** Raihana Nadra Alkaff, Taro Kamigaki, Mayuko Saito, Fajar Ariyanti, Dewi Utami Iriani, Hitoshi Oshitani

**Affiliations:** 1 Department of Virology, Tohoku University Graduate School of Medicine, 2-1, Seiryo-machi, Aoba-ku, Sendai, 980-8575 Japan; 2 Public Health Study Program, Faculty of Health Sciences, Syarif Hidayatullah State Islamic University Jakarta, Kampus 2, Jl. Kertamukti No.5, Ciputat, Tangerang Selatan, Banten 15419 Indonesia

**Keywords:** Antibiotics, Indonesia, Acute respiratory infection, Diarrhea, Child, Antimicrobial resistance

## Abstract

**Background:**

The incidence of antimicrobial resistance has been increasing worldwide in the past decades, which includes resistance to bacteria that cause common childhood illnesses, such as acute respiratory infections and diarrhea. Numerous children with those common illnesses are treated with antibiotics. However, in such cases, antibiotic treatment is not required. Community-based studies focusing on antibiotic use among children are still limited. This study aimed to identify the prevalence of antibiotic use for common childhood illnesses and to investigate factors associated with antibiotic use in children under 5 years old as well as female caregivers in a rural community in Indonesia.

**Methods:**

A cross-sectional study of 334 children in three villages of Banten Province, located in the western part of Java Island, was conducted in May 2018. Female caregivers who were responsible for providing medications to children were interviewed. We obtained information such as demographic data, any common clinical illness within the last 30 days, and antibiotic usage during an episode of illness. We excluded children with underlying disease that require a regular follow-up and children who were hospitalized in the last 30 days in the analysis. Antibiotic use answered by female caregivers was verified by checking its package or showing photos of various antibiotics to the female caregivers. Crushed antibiotics were confirmed with health professionals.

**Results:**

A total of 203 children had clinical symptoms, and the most common symptom was fever and respiratory symptoms. In total, 49.3% received antibiotics, and 66% of them were prescribed by private health professionals. Only two children received antibiotics without a prescription. The most common antibiotic used among children was amoxicillin.

**Conclusions:**

The high prevalence of antibiotic use was observed in children under 5 years of age, and the major source to obtain antibiotics was to consult health professionals. Training on appropriate antibiotic use must be conducted for health professionals in not only public but also private sectors.

## Background

The incidence of antimicrobial resistance (AMR) has been increasing worldwide in the past decades, which include resistance to bacteria that cause common infections in children, such as acute respiratory infections (ARI) and diarrhea [1–5]. Numerous children with ARI and diarrhea are treated with antibiotics. However, in these cases, antibiotic treatment may not be required [6–8]. Previous studies have shown that the use of antibiotic was associated with higher rates of resistance in children [9, 10].

A large variation was observed in the use of antibiotics among children between countries [11]. The global consumption of antibiotics had increased significantly, mainly due to the increase of antibiotic use in low- and middle-income countries [12]. Therefore, the appropriate use of antibiotics must be promoted in these countries to prevent the increasing trend of antibiotic resistance at the global level. However, in low- and middle-income countries, the inappropriate use of antibiotics is common [13–15]. Furthermore, the utilization of unprescribed antibiotics is a major issue particularly in low- and middle-income countries [16–18]. Studies about knowledge and practices of and attitudes toward the use of antibiotics have indicated that lower socioeconomic status and education level may be important factors for the misconceptions of antibiotic use among caregivers [19–21].

Despite numerous publications showing the prevalence of antibiotic use and its associated factors, community-based surveys focusing on children aged under 5 years are still limited in low- and middle-income countries, including Indonesia, where misuse and overuse of antibiotics are considered a more serious problem. The patterns of antibiotic use were studied in health care settings including primary care facilities [14, 22] and teaching hospitals [23]. A study conducted in Surabaya, Indonesia, has indicated that antibiotics without prescriptions are widely sold in pharmacies and kiosks [24]. Moreover, a community-based study in Yogyakarta has shown that self-medication with antibiotics was common in adult patients [25]. In 2015, the Ministry of Health of Indonesia had released the 2015–2019 strategic plans for implementing existing regulations and guidelines and rolling out of AMR-related activities. However, there is no national AMR surveillance currently implemented, and the data about AMR remains limited in the country [26–28]. Actions against AMR need to be further strengthened all over the country [26].

We conducted a study on antibiotic use in children under 5 years old in a rural community in Indonesia to identify the prevalence of antibiotic use for common childhood illnesses and to investigate factors associated with antibiotic use in children as well as female caregivers.

## Results

**Fig. 1:**
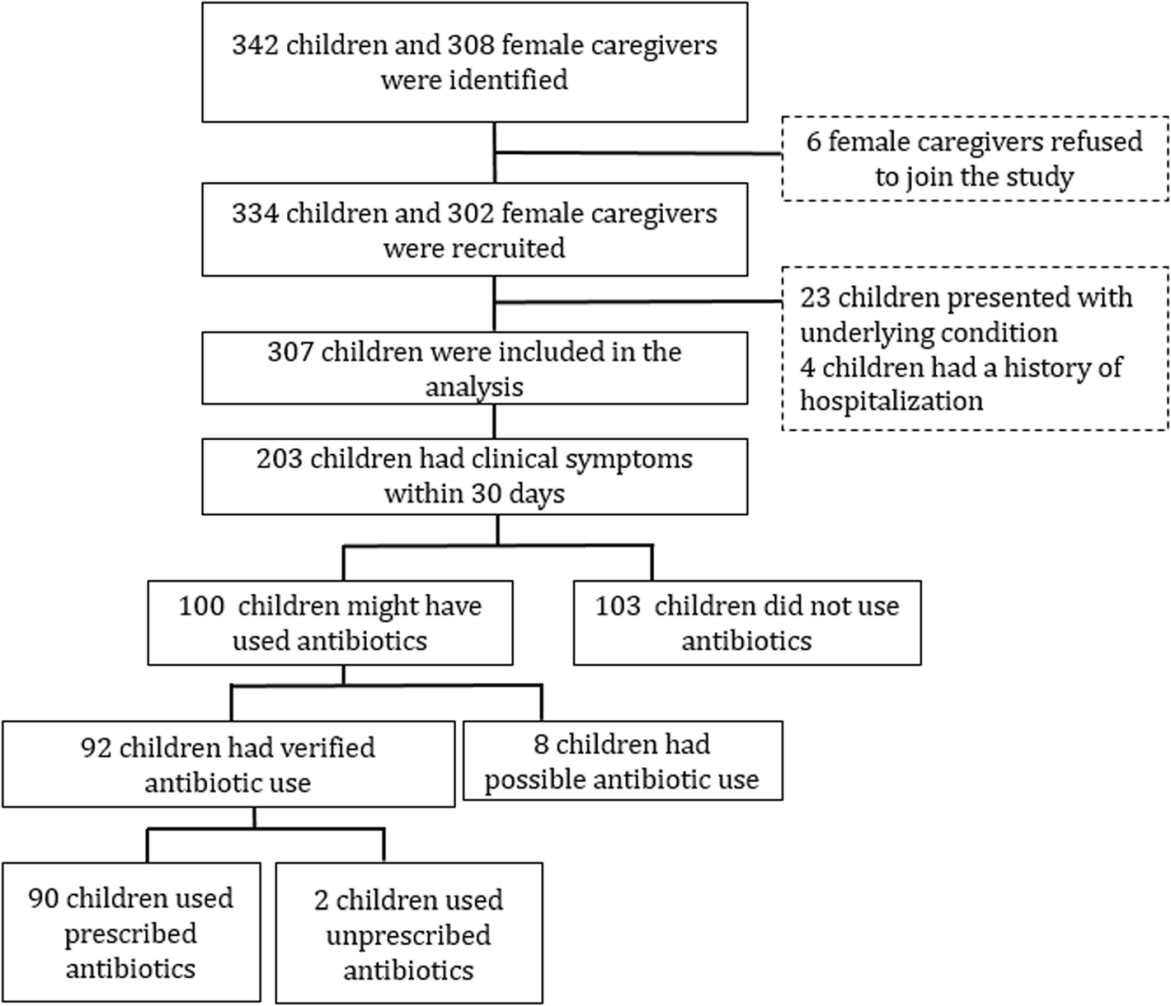
Flowchart of the recruitment of study participants

Out of 1645 children listed in the registration books at health-integrated posts, 342 children aged under 5 years in 308 households were selected for the study (Fig. 1). Out of 308 female caregivers, 302 (98.1%) agreed to participate in the study. There were 334 children in 302 households. Of these 334 children, 23 presented with underlying conditions, and four were hospitalized. These children were then excluded from the analysis. The remaining 307 children were included in the analysis. Of 307 children, 203 (66.1%) had at least one clinical symptom within the last 30 days.

### Sociodemographic characteristics of the female caregivers and children

Table 1 shows a summary of the sociodemographic characteristics of the female caregivers and children. The mothers of the children comprised over 94% of the female caregivers. The median age of the female caregivers was 30.3 years. About 60% of female caregivers had education at the primary school level or lower. Approximately 64% of households had an income lower than the minimum salary in Rangkasbitung district. About one third of the female caregivers claimed that their children had health insurance. The ages of the children were not equally distributed. That is, there is a higher proportion of older children. The proportion of boys was slightly higher than that of girls (56% vs. 44%).

**Table 1:** Characteristics of the female caregivers and children

Characteristics	*n* (%)
Female caregivers (*n* = 302)	
Age (years), median (IQR)	30.3 (25.3–35.7)
Relationship with the child	
Mother	285 (94.4)
Grandmother	10 (3.3)
Others	7 (2.3)
Two or more children aged < 5 years	31 (10.3)
Level of education	
None/primary	175 (57.9)
Secondary	86 (28.5)
Higher than secondary	41 (13.6)
Income of the family in the previous month^†^	
Lower than the minimum salary in Rangkasbitung district	193 (63.9)
Higher than the minimum salary in Rangkasbitung district	95 (31.5)
No answer	14 (4.6)
Living with extended family	139 (46.0)
With health insurance for child/children	104 (34.4)
Children (*n* = 334)	
Age in year	
< 1	44 (13.2)
1	32 (9.6)
2	96 (28.7)
3	53 (15.9)
4	109 (32.6)
Gender	
Male	187 (56.0)
Female	147 (44.0)

*IQR* Interquartile range

^†^The minimum salary in Rangkasbitung district in 2017–2018 was IDR 2,312,384 or $158.1

### Characteristics of children with any clinical signs and those who used antibiotics

The median age of 203 children who had clinical symptoms within the last 30 days was 2 years and 10 months, and 54.7% were boys. More than 90% of children who had respiratory symptoms, diarrhea, or fever visited a health facility. A total of 100 children took either verified or possible antibiotics (Table 2). This meant 49.3% of children with clinical symptoms or 32.6% of children included in the analysis took the drug. Of those who received antibiotics, 63 were verified to have taken antibiotics based on the remaining package (*n* = 44) or via the identification of the photos of antibiotics (*n* = 19) by the female caregivers. Of the 63 children whose antibiotic use was verified, 62 took syrup and one took tablets. A total of 29 children used crushed drugs, which the health care staff verified as antibiotics. However, the name of these crushed antibiotics was not identified. Among 100 children, 8 were considered to have received possible antibiotics as recalled by the female caregivers.

**Table 2:** Proportion of antibiotic use among children who developed clinical symptoms within the last 30 days (*n* = 203)

Characteristics	Antibiotic use		
	Total	Verified	Overall^†^
	*n*	*n* (%)	*n* (%)
Total	203	92 (45.3)	100 (49.3)
Health facility visit			
Visited	186	90 (48.4)	98 (52.7)
Did not visit	17	2 (11.8)	2 (11.8)
Symptoms^††^			
R + D + F	37	22 (59.5)	23 (62.2)
R + D	2	1 (50.0)	1 (50.0)
R + F	100	46 (46.0)	48 (48.0)
R	20	5 (25.0)	6 (30.0)
D + F	4	2 (50.0)	2 (50.0)
D	4	1 (25.0)	1 (25.0)
F	36	15 (41.7)	19 (52.8)
Age			
< 1 year	35	19 (54.3)	21 (60.0)
1 year	23	11 (47.8)	13 (56.5)
2 years	54	22 (40.7)	24 (44.4)
3 years	32	17 (53.1)	18 (56.3)
4 years	59	23 (39.0)	24 (40.7)

^†^Overall antibiotic use is a combination of verified and possible antibiotic use

^††^*R* Respiratory symptoms (cough or difficulty of breathing), *D* Diarrhea (frequent loose or liquid stool). *F* Fever (fever or feverish)

The name of the antibiotics used in 63 children was identified, of whom 53 (84.1%) used amoxicillin. Other antibiotics included trimethoprim and sulfamethoxazole (*n* = 4), cefadroxil (*n* = 4), chloramphenicol (*n* = 1), and metronidazole (*n* = 1).

Data about the proportion of verified, and possible antibiotic use by health facility visit, symptoms, and age are shown in Table 2. Most children (98.0%) received antibiotics from health facilities. Seventeen children did not visit any of the health facilities. Among them, only two (11.8%) took leftover antibiotics at home without a prescription. Proportions of antibiotic use varied among children with different clinical symptoms (Table 2). The highest proportion (62.2%) of antibiotic use was observed in children who had a combination of respiratory symptoms, diarrhea, and fever, whereas the lower proportion of antibiotic use was observed in those with respiratory symptoms only and diarrhea only (30.0% and 25.0%, respectively). Children aged under 1 year had the highest proportion (60.0%) of antibiotic use. However, the proportions were also high in older children ranging from 40.7 to 56.5% (Table 2).

Children who visited the clinic of a private doctor had a higher proportion of antibiotic use (60.0%) than those who visited primary health centers (51.6%). However, the result was not statistically significant (Table 3). Similarly, the odds ratio (OR) for taking antibiotics at the private clinics of midwives/nurses was lower than that at private doctor’s clinics though there was no statistical significance. Children who did not visit the health facility had the lowest proportion of antibiotic use (11.8%), and the differences were statistically significant when compared with children who visited one of the health facilities (*p* < 0.001).

**Table 3:** Association between antibiotic use and the type of health facility visited, clinical symptoms and age of children (*n* = 203)

Characteristics	Description	Total *n*	Antibiotic use		aOR^††^ (95% CI)	*p* value
			Yes^†^ *n* (%)	No *n* (%)		
Type of health facility visited	Private doctor clinic	55	33 (60.0)	22 (40.0)	Ref.	–
	Private midwife/nurse clinic	69	33 (47.8)	36 (52.2)	0.42 (0.20–1.04)	0.18
	Primary health centers	62	32 (51.6)	30 (48.4)	0.64 (0.29–1.59)	0.36
	Did not go to any health facility	17	2 (11.8)	15 (88.2)	0.08 (0.01–0.54)	< 0.001*
Clinical symptoms						
Fever	No	26	8 (30.8)	18 (69.2)	Ref.	
	Yes	177	92 (52.0)	85 (48.0)	2.48 (0.97–7.03)	0.06
Respiratory symptoms	No	44	22 (50.0)	22 (50.0)	Ref.	
	Yes	159	78 (49.1)	81 (50.9)	0.88 (0.44–1.98)	1.00
Diarrhea	No	156	73 (46.8)	83 (53.2)	Ref.	
	Yes	47	27 (57.4)	20 (42.6)	1.83 (0.89–4.32)	0.24
Age of the child	< 2 years	58	34 (58.6)	24 (41.4)	Ref.	
	2–4 years	145	66 (45.5)	79 (54.5)	0.57 (0.30–1.25)	0.12

*aOR* adjusted odds ratio, *95% CI* confidence interval, *Ref*. Reference

*P* values for the type of health facility visited were assessed between a private doctor’s clinic and each of the other category

^†^The category “Yes” includes both verified and possible antibiotic use

^††^Adjusted for age of the child and living with extended family, or living with extended family only (for age of the child)

*p value < 0.05

When comparing children with and without a certain symptom (i.e., fever, respiratory symptoms, and diarrhea) (Table 3), no difference was observed between children with and without respiratory symptoms. Children with fever had a higher proportion of antibiotic use (52.0%) than those without fever (30.8%). However, the result was not statistically different (*p* = 0.06). Moreover, no difference was noted in the proportion of antibiotic use between children aged less than 2 years, and those aged 2–4 years (Table 3).

In Table 4, we compared the characteristics of female caregivers as well as children who used and did not use antibiotics. The female caregivers who did not live with extended family had a significantly higher proportion of antibiotics use (57.7% vs. 40.4%, *p* = 0.014) and odds ratio to have antibiotics was also significantly low among those who live with extended family (OR = 0.47, 95% CI = 0.25–0.97). No difference was observed in the proportion of antibiotic use in terms of the other characteristics of the female caregivers (relationship with the child, age group, educational level, family income, and with health insurance for children) (Table 4).

**Table 4:** Association between antibiotic use and characteristics of female caregivers (*n* = 203)

Characteristics	Description	Total *n*	Antibiotic use		aOR^††^ (95% CI)	*p* value
			Yes^†^ *n* (%)	No *n* (%)		
Relationship with the child						
Mother		192	94 (49.0)	98 (51.0)	Ref.	0.91
Grandmother		7	4 (57.1)	3 (42.9)	1.42 (0.24–11.68)	
Others		4	2 (50.0)	2 (50.0)	0.77 (0.14–5.77)	
Age of female caregivers	< 30 years	114	58 (50.9)	56 (49.1)	Ref.	
	≥ 30 years	89	42 (47.2)	47 (52.8)	0.83 (0.45–1.71)	0.60
Educational level of female caregiver	None or primary	91	41 (45.1)	50 (54.9)	Ref.	
	Secondary or above	112	59 (52.7)	53 (47.3)	1.04 (0.56–2.11)	0.28
Lower than the minimum salary in Rangkasbitung district	No	64	32 (50.0)	32 (50.0)	Ref.	
	Yes	130	65 (50.0)	65 (50.0)	0.96 (0.51–2.03)	1.00
Living with extended family	No	104	60 (57.7)	44 (42.3)	Ref.	
	Yes	99	40 (40.4)	59 (59.6)	0.47 (0.25–0.97)	0.01*
With health insurance for child(ren)	No	133	70 (52.6)	63 (47.4)	Ref.	
	Yes	69	29 (42.0)	40 (58.0)	0.90 (0.48–1.89)	0.15

*aOR* adjusted odds ratio, *95% CI* confidence interval, *Ref.* Reference

The minimum salary in Rangkasbitung was compared with the household income in last month

^†^The category “Yes” includes both verified and possible antibiotic use

^††^Adjusted for age of the child and living with extended family, or age of the child only (for living with extended family)

*p value < 0.05

## Discussion

In the present study, about 33% of children received antibiotics within the last 30 days (about four courses per child per year), which is similar to the rate of antibiotic courses provided to children less than 2 years (4.9 courses per child per year) in a prospective cohort study conducted in eight low- and middle-income countries [11]. About half of the children presented with common clinical symptoms, including fever, respiratory symptoms, and diarrhea, received antibiotics. The Demographic and Health Survey of Indonesia conducted in 2012 has also shown that the prevalence of antibiotic use ranged from 12.5% for diarrhea to 39% for ARI that occurred within 2 weeks before the survey [29].

The Integrated Management Childhood Illness (IMCI) was developed by the World Health Organization (WHO) and the United Nations International Children’s Emergency Fund (UNICEF) to guide the management of common childhood illnesses in resource-limited settings [30]. Indonesia has adopted IMCI and has been implementing it nationwide [31]. Amoxicillin must be taken when the diagnosis of pneumonia, as characterized by rapid breathing, is made. We did not assess the severity of the condition of the children with ARI in this study; however, most ARI cases reported in this study might be mild, which do not require antibiotic treatment partly because we excluded hospitalized cases in the analysis. A previous study in low-middle-income countries also reported 44% of acute respiratory illness were treated with antibiotics [11].

Similarly, only acute diarrhea with bloody stool or suspected cholera cases requires oral antibiotic treatment, such as cotrimoxazole and tetracycline. Whether diarrhea was bloody or suggestive of cholera was not assessed in this study. However, Indonesia reported low cholera incidence in the last decade [32] and dysentery was frequently observed in the rainy season (December to January) [33]. The proportion of the symptoms mentioned above is considered to be low among all diarrhea cases during the non-epidemic period. Therefore, most antibiotics provided to children with ARI and acute diarrhea might not be necessary. Although no statistical significance was observed, odds for taking antibiotics was the highest in comparing children with fever and those without. When children who had ARI with and without fever were compared, a higher proportion of antibiotic use was observed in those with ARI and fever than in those with ARI without fever. A previous study in low- and middle-income countries reported that fever was independently associated with antibiotics treatment [11]. Health facilities in the study sites also might be using fever as criteria to give antibiotics.

Only two children received unprescribed antibiotics. On the contrary, another community-based study in Indonesia showed that more than half of the antibiotics used were not prescribed [25]. However, this study was conducted for the adult population in 2010. There might be different patterns of non-prescribed antibiotic use between children and adults. The recent strict enforcement of regulations to limit the over the counter availability of antibiotics might have reduced the prevalence of non-prescribed use of antibiotics [26, 34]. Continuous studies must be conducted to monitor the trend of using non-prescribed antibiotics in different age groups.

Although most children received antibiotics from health care facilities with the prescription, the overuse of antibiotics in health care facilities is a major concern, as reported in previous studies in Indonesia [14, 22]. About two thirds of antibiotics were prescribed by private doctors or private midwives/nurses. IMCI has not been fully implemented even in primary government health centers due to various challenges, including the shortage of trained staff [35]. It would be more difficult to train health care professionals, such as midwives, in the private clinic.

The most common antibiotic used among children in the present study was amoxicillin, which is the first-line treatment recommended for pneumonia in the IMCI in Indonesia in 2015. Amoxicillin is not recommended for children with acute diarrhea. However, it was commonly provided to children with diarrhea in the present study. Recently, the WHO classifies antibiotics in the WHO essential medicines list into three groups: Access, Watch, and Reserve (AWaRe) [36]. Access antibiotics are first-line or second-line antibiotics for common infectious diseases. Most of the antibiotics used for children in the present study were Access antibiotics. Antibiotics in the Watch list, such as third-generation cephalosporins and quinolones were not prescribed even by private doctors. However, further training on the proper use of antibiotics must be conducted for health professionals in both the public and private sectors.

The sample size of the study was not enough to obtain data about the association between the characteristics of female caregivers and antibiotic use. Age, educational level, family income, and health insurance for children were not significantly associated with antibiotic use. However, a higher proportion of antibiotics use was observed in female caregivers who did not live with extended family than in those who lived with extended family. The reason for such difference was not clear. A caregiver living with extended family might obtain more appropriate advice from other family members.

The present study had some limitations. First, we collected information about symptoms and antibiotic use from female caregivers. Although we only assessed the episodes within the last 30 days before the interview, responses of the caregivers were still subject to recall bias. Second, we could not validate antibiotic use in some cases or specify types of some crushed antibiotics. Third, it was not possible to classify ARI into different severity categories, including pneumonia, and bloody diarrhea and suspected cholera were not identified among diarrhea cases. Thus, we could not know how many children with ARI and diarrhea required antibiotic treatment. Despite these limitations, results from the present study provided important insights for future interventions and data about antibiotic use in children in the rural Indonesian community.

## Conclusion

The high prevalence of antibiotic use was observed in children under 5 years of age with common clinical symptoms such as fever, respiratory symptoms, and diarrhea. The major source to obtain antibiotics was to consult health professionals, and 66% of antibiotics were from private doctors, midwives, or nurses. Training on appropriate antibiotic use must be conducted for health professionals in not only public but also private sectors.

## Methods

### Study setting and design

**Fig. 2:**
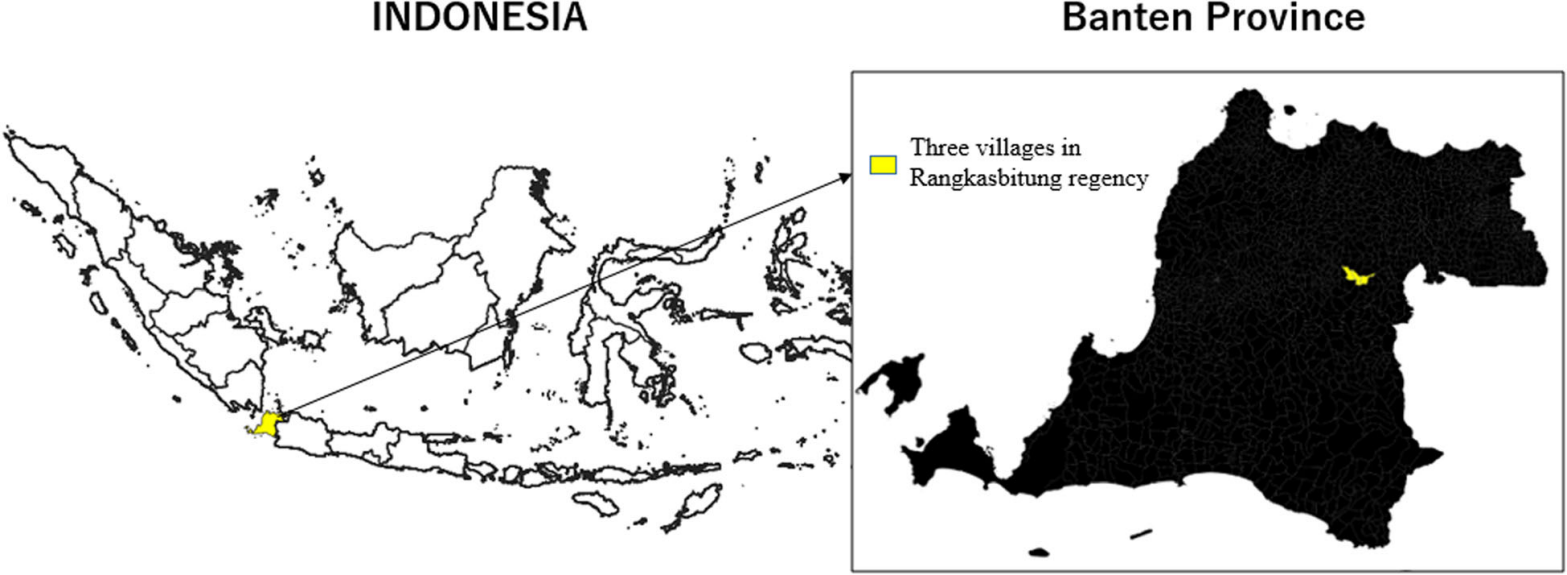
Map of the study area

A cross-sectional survey was conducted in May 2018 in Rangkasbitung district, Banten Province, located in the western part of Java Island, Indonesia (Fig. 2). The total population in Rangkasbitung district was 122,646 in 2016 [37]. The main industry of the district is agriculture. Rangkasbitung district is reported as a non-endemic malaria area; thus, antimalaria treatment is not recommended [31]. In Indonesia, the primary health center is called puskesmas, which covers a catchment of approximately 30,000 people [38]. A health center comprised several health professionals, including physicians, nurses, and midwives. There are lower level facilities under each primary health center, including the village health post (poskesdes) and the health-integrated posts (posyandu) [39]. The former is a facility with at least one midwife, and basic health services such as family planning, maternal care, and immunization are provided [40]. The latter is mainly operated by volunteer health workers (cadres) with occasional support from midwives, and anthropometry for children under five years old, family planning, immunization, nutrition, and sanitation programs are provided [41]. We selected all three villages (desa) in the catchment area of Mekarsari primary health center. There is one village health post and 24 health-integrated posts under the center.

### Participants of the study

Our sample size was simply calculated by assuming an expected prevalence of antibiotic use of 25% with 5% precision and 5% level of significance. We also considered 5% as the non-response rate in the survey to have a target sample size of 300 children. We stratified listed children by age group (< 1 year, 1–2 years, and 3–4 years). The number of children in each stratum was proportionally allocated to the number of children registered in each post. We conducted a face-to-face household interview with a female caregiver, which was defined as a female household member who was responsible for providing medications to children. When the female caregiver was not around, we visited the household again until a maximum of three visits. If we could not meet the female caregiver within three visits, the alternative child in the list was included in the study. We identified and interviewed one female caregiver in each household when two or more female caregivers lived in the same household.

### Data collection

A standardized questionnaire was developed before the survey, which consisted of two parts. The first part was for the female caregivers that included demographic characteristics, level of education, and household income, and the other part was for children that included illness history and antibiotic usage during an episode. The questionnaires and informed consent form were initially developed in English and then translated into local languages (Bahasa and Sundanese). The questionnaire was applied to the ESRI Survey 123 (ESRI Inc., Redlands, CA) for inputting data during the interview. Field staffs obtained informed consents from female caregivers before the interview. The regional minimum salary (Upah Minimum Regional/UMR) was used as a cut-off to assess the economic status of the household [42, 43]. We interviewed female caregivers about the presence of clinical signs among children within 30 days before the interview. The data included fever or feverish (fever), cough or difficulty of breathing (respiratory symptoms), and frequent loose or liquid stools (diarrhea). If there were more than one illness episodes within 30 days before the interview, we collected the information related to illness episode including antibiotics use only about the most recent episode.

We used three steps to verify antibiotic use and to identify the type of antibiotics provided to children. First, female caregivers were asked to show a drug package or bottle to the interviewers if available. Second, if package or bottle was not available, the photos of various antibiotics commonly used in the study area were shown to the female caregivers to identify the antibiotics that were provided. If the types of antibiotics were not verified using these steps, we asked them if they recalled the name of the drug. If the name of the drug mentioned by the female caregivers was that for antibiotics, we categorized it as might have used antibiotics. In the study area, children sometimes receive antibiotics in a crushed powder (puyer). If the crushed powder was provided, we checked the medical records at the health center or contacted to health professionals who prescribed them. If antibiotic use was confirmed based on the information obtained from the medical records or health professionals, we categorized them as verified use of antibiotics.

### Data analysis

We excluded children from the analysis of antibiotic use if female caregivers stated that the child had any disease or condition that requires a regular medical follow-up, or was hospitalized because of the illness within the last 30 days. We defined health center as any health facility under the government system, including primary health center, village health post, and health-integrated post. Prevalence of antibiotic use was calculated as the proportion of the number of children who received antibiotics (both verified and possible antibiotics) to the number of children with clinical symptoms. The characteristics of children who used and did not use antibiotics and the female caregivers were compared using the chi-square test. The statistically significant level was set at less than 5% (*p* < 0.05). The association between antibiotic use and the type of health facility visited, clinical symptoms, age, and characteristics of the female caregivers were evaluated using logistic regression with generalized estimating equations to account for the effect of having the same female caregivers. Odds ratios were then adjusted with two variables that showed *p* value < 0.1 in the univariate analysis: age of children and living with extended families or not. Extended family was defined as a family that extends beyond the nuclear family to include other relatives. To test statistical significance, 95% confidence intervals (CI) were calculated. The analysis was performed using either IBM SPSS Statistics for Windows, Version 21.0 (IBM, Armonk, NY) or Microsoft R Open 3.5.1 (Microsoft and R core team, Redmond, WA) with geepack package.

## Data Availability

The datasets used and/or analyzed during the current study are available from the corresponding author on reasonable request.

## Abbreviations

AMR: Antimicrobial Resistance
ARI: Acute Respiratory Infections
AWaRe: Access, Watch, and Reserve
IMCI: Integrated Management of Childhood Illness
Poskesdes: *Pos Kesehatan Desa*
Posyandu: *Pos Pelayanan Terpadu*
Puskesmas: *Pusat Kesehatan Masyarakat*
UMR: Upah Minimum Regional
UNICEF: the United Nations International Children’s Emergency Fund
WHO: World Health Organization
